# Effects of Aluminosilicate Gel Treatment and TiO_2_ Loading on Photocatalytic Properties of Au–TiO_2_/Zeolite Y

**DOI:** 10.3390/gels9060503

**Published:** 2023-06-20

**Authors:** Gabriela Petcu, Florica Papa, Elena Maria Anghel, Irina Atkinson, Silviu Preda, Simona Somacescu, Daniela C. Culita, Adriana Baran, Elena Madalina Ciobanu, Luiza Maria Jecu, Mariana Constantin, Viorica Parvulescu

**Affiliations:** 1 Institute of Physical Chemistry “Ilie Murgulescu” of the Romanian Academy, 202 Splaiul Independentei, 060021 Bucharest, Romaniamanghel@icf.ro (E.M.A.); iatkinson@icf.ro (I.A.); mciobanu@icf.ro (E.M.C.); 2 National Institute for Research & Development in Chemistry and Petrochemistry-ICECHIM, Spl. Independentei 202, 060021 Bucharest, Romania

**Keywords:** aluminosilicate gel, zeolite Y, Ti–Au zeolite Y, photocatalysis, surface plasmon resonance, effect of TiO_2_ loading, photodegradation, amoxicillin

## Abstract

The present work reports the synthesis of efficient Ti–Au/zeolite Y photocatalysts by different processing of aluminosilicate gel and studies the effect of titania content on the structural, morphological, textural, and optical properties of the materials. The best characteristics of zeolite Y were obtained by aging the synthesis gel in static conditions and mixing the precursors under magnetic stirring. Titania (5, 10, 20%) and gold (1%) species were incorporated in zeolite Y support by the post-synthesis method. The samples were characterized by X-ray diffraction, N_2_-physisorption, SEM, Raman, UV–Vis and photoluminescence spectroscopy, XPS, H_2_-TPR, and CO_2_-TPD. The photocatalyst with the lowest TiO_2_ loading shows only metallic Au on the outermost surface layer, while a higher content favors the formation of additional species such as: cluster type Au, Au^1+^, and Au^3+^. A high TiO_2_ content contributes to increasing the lifetime of photogenerated charge careers, and the adsorption capacity of the pollutant. Therefore, an increase in the photocatalytic performances (evaluated in degradation of amoxicillin in water under UV and visible light) was evidenced with the titania content. The effect is more significant in visible light due to the surface plasmon resonance (SPR) effect of gold interacting with the supported titania.

## 1. Introduction

The importance of faujasite-based catalysts has led to a growing interest in facile synthesis procedures that allow for preparing high-performance materials with improved catalytic properties. The performance of zeolites is greatly influenced by their size and shape, morphological homogeneity being desirable [1,2,3]. It is well known that in zeolite synthesis, the mixing of aluminate and silicate in a high pH medium led to the formation of hydrogel. This behavior was explained by the spontaneously bonding in a first stage of some aluminate ions to the silica oligomers, following multiple processes of dissolution and regeneration of the gel until the equilibrium state is reached [4]. Previous studies evidenced the importance of the aging process on gel chemistry in the synthesis of zeolite materials by controlling the nucleation and crystal growth [5]. Finding the parameters that determine the crystal morphology and size is essential in tailoring the properties of zeolites [1] which are often used as supports in obtaining photocatalysts because of their specific advantages such as structure, stability, high surface area, uniform pores, and channels with high adsorption capacity, acid-basic properties, ion exchange ability, and electric field of the framework [6,7,8].

TiO_2_ is a widely studied semiconductor due to its well-known advantages and potential application in different fields such as solar energy conversion, antibacterial treatments, and heterogeneous photocatalysis [9,10,11,12]. However, TiO_2_ applications are limited because of its large energy band gap and the tendency of particles to agglomerate in aqueous media with a reduction in the exposed surface area. The narrowing of the energy band gap was achieved by TiO_2_ doping with various metals or nonmetal species [13,14,15,16,17,18]. A method to diminish agglomerations is the immobilization of TiO_2_ nanoparticles on a support framework, such as zeolites [19,20,21,22]. In this case, the surface chemistry of the support plays a decisive role in photocatalytic activity and stability [23]. The photocatalysts thus obtained showed, in most cases, a higher activity than unsupported TiO_2_, assigned to the synergism between the electron-rich nature and large surface area of the zeolites which facilitates an excellent dispersion of TiO_2_ nanoparticles, to adsorption of organic substrates on the zeolite support and to the effective separation of the photogenerated electrons and holes by the electric field of the zeolite framework [24]. The adsorption of pollutants on the photocatalyst is a very important step because charge carriers resulted under irradiation have a short lifetime, so the presence of pollutant molecules adsorbed on the surface have a higher probability to interact with the active sites. A significant problem of TiO_2_-loaded zeolite is the structural collapse of the zeolite structure when the optimum TiO_2_ loading is exceeded. Thus, TiO_2_/zeolite Na-A catalyst was successfully synthesized by a slightly modified sol–gel technique and the effects of TiO_2_ loading on structural, optical, adsorptive, and photocatalytic properties were evaluated [6]. The obtained results showed a small effect on the zeolite structure, decreasing of band gap energy with TiO_2_ loading, and increasing photocatalytic activity.

The Au–TiO_2_ system has attracted considerable attention because of the high and stable photocatalytic activity that can be achieved under both UV and visible light excitation [25]. TiO_2_ was considered to be a good support for Au photocatalysts due to the strong interaction metal-support, chemical stability, and acid-base properties [26]. The main role of TiO_2_ utilized in photocatalysis is to generate, under light irradiation, charge carriers and reactive oxygen species (ROS). The gold nanoparticles influence the activation of TiO_2_ by plasmonic resonance energy transfer or by “hot electrons” injected into the conduction band of the TiO_2_ support [27]. The surface and interface properties of plasmonic Au/TiO_2_ nanocatalysts play a critical role in determining their performance in photocatalytic oxidation reactions [28,29].

The previous studies showed that the dispersion of titanium species and gold significantly influences the catalytic activity, durability, and selectivity in amoxicillin photodegradation [20]. These properties were significantly influenced by the support surface area, porous structure, and morphology which have modulated the electronic Au–TiO_2_ interaction. The synergistic effect between Au–TiO_2_ and supports was the result of an intimate contact between components, determined by the dispersion of titania, and implicitly of gold species on the support. 

In this study, new active photocatalysts were obtained by the manufacturing of aluminosilicate gel to obtain the zeolite Y with a crystalline structure, and its modification with various amounts of TiO_2_ (5, 10, 20%) and gold (1%) species. For this purpose, the effects of the experimental conditions during the processing of the synthesis gel on the structural and morphological properties of zeolite Y were evaluated. Previous studies showed the formation of zeolite Y crystalline phase during the sol–gel process [30]. Compared to the other porous supports used (MCM-48, KIT-6, SBA-15, hierarchical zeolite Y), microporous zeolite Y with the same concentration of TiO_2_ (10%) seems to have a lower activity [20]. This is probably the reason why Au–TiO_2_ photocatalysts supported on zeolite Y have been less studied. In this context, the present work aimed to evaluate the effect of TiO_2_ loading on the interaction with the subsequently immobilized gold species (especially the optical properties) in order to improve the photocatalytic activity of these materials. For the new samples obtained, the test reaction was also amoxicillin photodegradation. 

## 2. Results and Discussion

### 2.1. Effect of Aluminosilicate Gel Treatment 

In order to obtain high-performance materials for photocatalytic applications, the hydrogel spontaneously obtained after mixing aluminate and silicate was differently treated. Thus, the influence of the reactants mixing method (magnetic/assisted by ultrasound) and the effect of stirring during the hydrogel aging were studied. Structural and morphological changes that occur by varying the experimental conditions during the aging of aluminosilicate hydrogel used to synthesize zeolites Y were investigated by X-ray diffraction, Raman spectroscopy, and scanning electron microscopy.

#### 2.1.1. XRD Analysis

**Figure 1:**
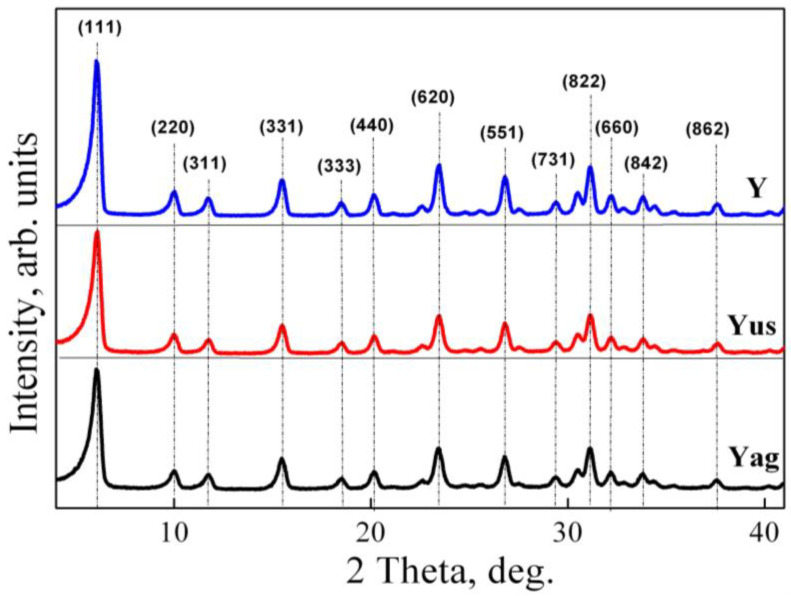
Wide-angle X-ray diffraction patterns of zeolite Y obtained by different treatments of aluminosilicate hydrogel during the aging step.

The X-ray diffraction patterns recorded for the powders obtained by different gel treatment procedures are summarized in Figure 1. The detectable diffraction peaks correspond to (111), (220), (311), (331), (333), (440), (620), (551), (731), (822), (660), (842), and (862) planes of the typical structure of faujasite Na-Y (ICDD 00-038-0239) [31]. The changes in intensity depending on the treatment applied to the synthetic gel indicate some variation in the crystal structure. Thus, the mixing of the reactants under magnetic stirring and aging of the aluminosilicate gel in the static mode (the second route, as described in the sample preparation section) led to the formation of zeolite Y with the highest degree of crystallinity.

#### 2.1.2. Raman Spectroscopy

**Figure 2:**
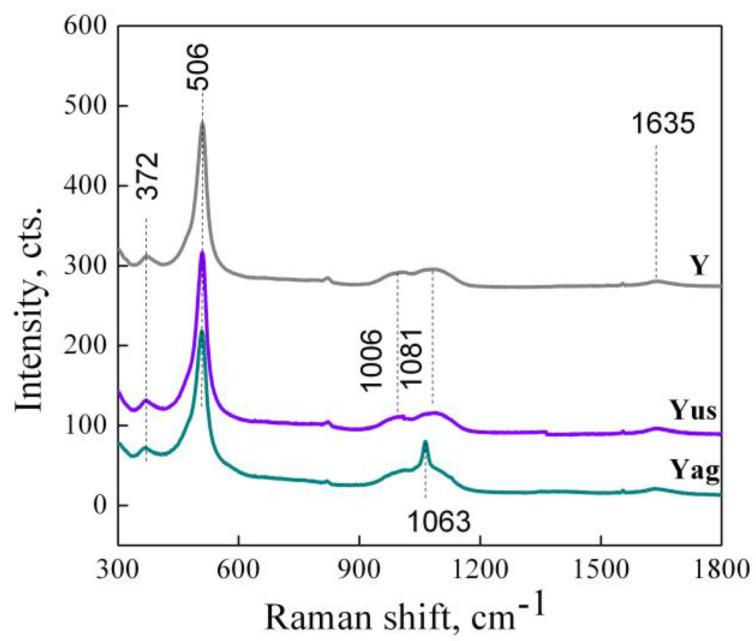
UV–Raman spectra of the zeolite materials obtained by different treatment of aluminosilicate gel.

The UV–Raman spectra of the zeolite powders obtained by different treatments of aluminosilicate gel are illustrated in Figure 2. For all the samples, the spectral features of zeolite Y at 372, ~500, 1006, and 1081 cm^−1^ were noticed, assigned to bending vibrations of the six- and four-membered SiO_4_ rings and asymmetric stretching mode of the T-O linkages (T stands for Al and/or Si) [32]). The asymmetric stretching modes of T-O bonds at 1063 cm^−1^ in the Y_ag_ spectrum are intermediate to the ones for X zeolite (1075 cm^−1^) and Y zeolite (1055 cm^−1^) [32] pointing out that the Si/Al ratio could be slightly modified in the Y_ag_ sample. Analogous spectral features to Y were depicted for the Y_us_ sample. This is probably due to the entrainment of more aluminosilicate by stirring during aging which leads to a slight change in the Si/Al ratio.

#### 2.1.3. SEM Images

**Figure 3:**
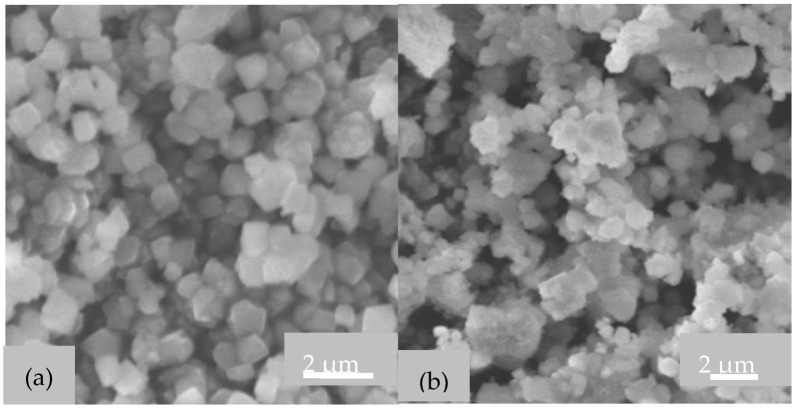

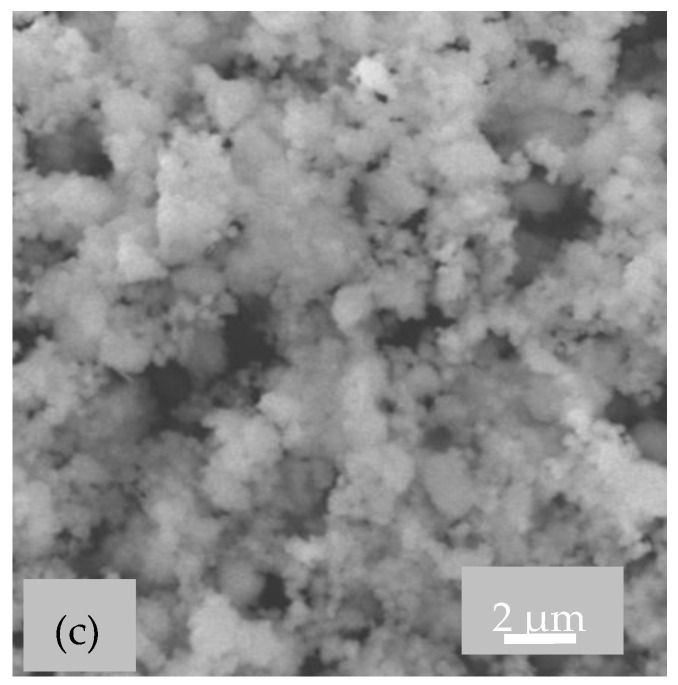
SEM images of zeolite (**a**) Y, (**b**) Yus, (**c**) Yag.

SEM images of zeolite Y powders obtained by different treatments of synthesis gel are illustrated in Figure 3. 

It is obvious that the morphology of zeolite Y powders can be controlled by manufacturing the aluminosilicate hydrogel. Thus, octahedral particles, with smooth faces, specific to zeolite Y were obtained using the classical stirring method and aging the gel in the static mode. Smaller crystals were obtained in the case of gel aging under stirring (Figure 3b), due to the fact that accelerated mass transfer led to a rapid supersaturation [5]. Furthermore, an inhomogeneous morphology with slightly rounded particles and some agglomerates were noticed. These morphologies correspond to a lower degree of crystallinity, as was also highlighted with the help of the X-ray diffraction patterns shown in Figure 1.

### 2.2. Effect of TiO_2_ Loading

The results presented in Section 2.1 pointed out the possibility to design the aluminosilicate hydrogel by changing the experimental conditions, leading to obtaining faujasite Y materials with suitable properties for photocatalysis. Further, it was chosen the material obtained by the second route (see the representation illustrated in the sample preparation section), modified with different TiO_2_ loadings (5%, 10%, 20%) and Au (only 1%) reactive species and characterized by various techniques.

#### 2.2.1. XRD Analysis

**Figure 4:**
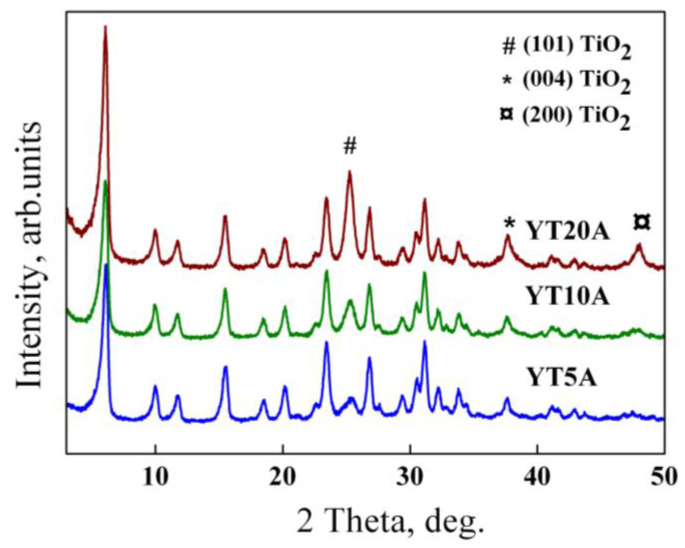
Wide-angle X-ray diffraction patterns of the prepared Au–Ti samples with different TiO_2_ loading and corresponding zeolite Y support.

Wide-angle X-ray patterns recorded for the synthesized photocatalysts are illustrated in Figure 4. It can be noticed that all three samples with different titania loading and 1% Au have comparable diffraction patterns with the zeolite support, suggesting preservation of the crystalline structure of microporous zeolite Y after Ti and Au incorporation.

For all three samples (YT5A, YT10A, YT20A) was observed the presence of three distinct diffraction lines at 2θ ≈ 25.2°, 37.8°, and 48.1°, corresponding to (101), (004), and (200) crystal planes of anatase TiO_2_ phase (Figure 4). Increasing titania loadings on zeolite Y support (5%, 10%, 20%) caused an increase in anatase diffraction line intensities. The average crystallite size, estimated using the Scherrer equation along the (101) direction, follows a similar trend (Table 1).

No diffraction peaks assigned to the gold-based compounds were identified in the X-ray diffractograms of the samples. It can be explained by the high dispersion of gold nanoparticles and also by their low relative concentration (<1%), below the detection limit of the instrument [33]. However, the presence of gold species together with titanium was highlighted by means of EDS analysis for YT10A sample (see Figure S1C from Ref. [20]).

#### 2.2.2. Textural Analysis

**Figure 5:**
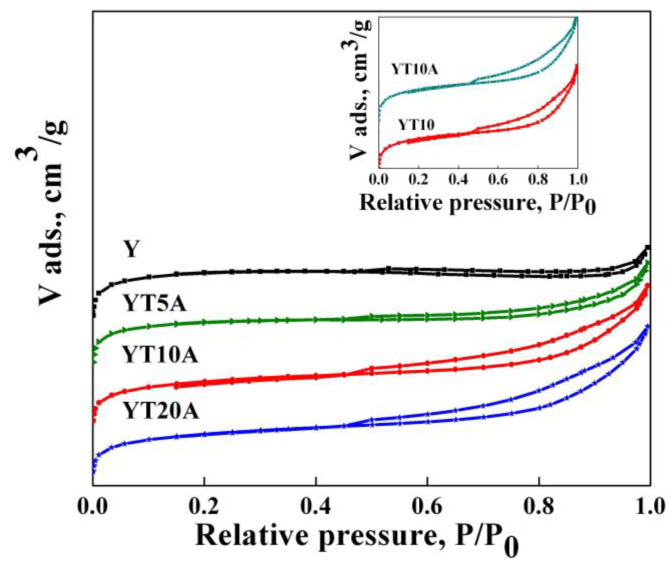
The nitrogen adsorption–desorption isotherms of the synthesized samples.

The N_2_ adsorption−desorption isotherms of the Ti–Au photocatalysts are presented in Figure 5. According to the IUPAC classification, the synthesized samples show a combination of type I and IV isotherms with H3 and H4 hysteresis loops [34]. In contrast to the case of using ordered mesoporous silica as support for the synthesis of Ti–Au photocatalysts, studied previously by our group [20], for which the shape and pores dimensions were preserved after titanium and gold immobilization, in the present work was observed a hysteresis loop came out after TiO_2_ immobilization. It can be explained by the formation of mesopores among TiO_2_ nanoparticles supported on microporous zeolite Y. BET surface areas, pore volume, and size after each impregnation step are summarized in Table 1. High values of the specific surfaces were obtained compared to other similar cases reported in the literature. For example, the BET surface area recorded for YT10A sample was 699 m^2^/g, unlike values such as 462 and 561 obtained for photocatalytic materials based on USY with 12% TiO_2_ and 3% gold [35]. Generally, a gradual decrease in BET surface area was noticed after titania loadings, while further immobilization of gold led to a slight increase in BET surface area.

#### 2.2.3. SEM Images

**Figure 6:**
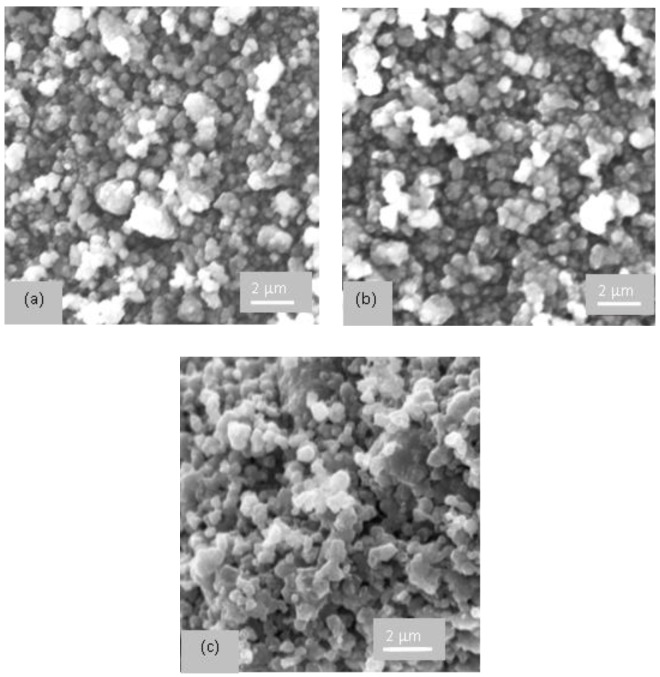
SEM images of (**a**) YT5A, (**b**) YT10A, and (**c**) YT20A photocatalysts.

The morphology of the synthesized samples was investigated by scanning electron microscopy, and the obtained images are presented in Figure 6. No change of morphology was observed after the impregnations of supports. Thus, for a high concentration of TiO_2_ (20%), YT20A sample, the preservation of octahedral morphology with smooth surfaces, specific to zeolite Y, and no aggregates or any other defects were observed. 

#### 2.2.4. XPS Results

XPS spectroscopy indicates low concentrations of gold on the sample surface (<1%), as the recorded photoelectron spectra are very noisy, even though there was a large number of runs recorded during data acquisition. Moreover, the Au4f spectra have a “band-like” shape which suggests the presence of different gold species accommodated under the experimental spectra envelope.

**Figure 7:**
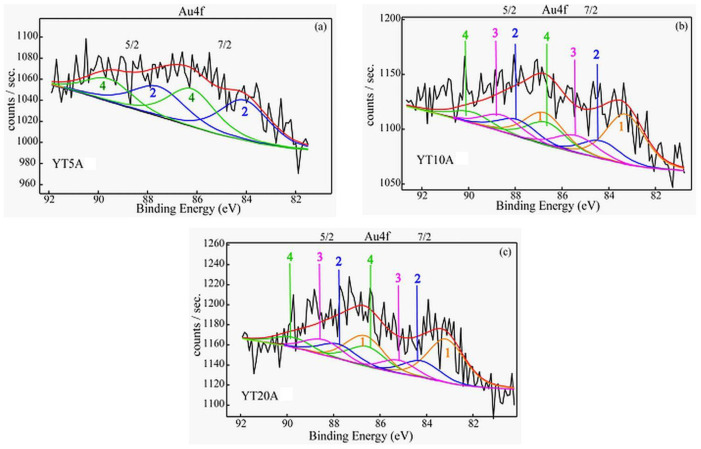
(**a**–**c**) Au 4f (7/2, 5/2) high resolution, deconvoluted XPS spectra for the synthesized samples.

The Au4f spectra recorded for the samples (Figure 7) showed the presence of different types of gold species depending on TiO_2_ loading. 

The interaction between gold nanoparticles and titanium species varied with the amount of immobilized TiO_2_. Thus, the sample with the lowest TiO_2_ content (YT5A) shows only Au metallic nanoparticles and Au^3+^ ions on the outermost surface layer. For a higher concentration of TiO_2_ (YT10A and YT20A samples), very small Au clusters (Au NCs) as well as Au^1+^ ions were additionally identified [36,37]. It is worth outlining that for the samples with higher TiO_2_ content (10, 20%) the relative concentrations of the aforementioned species do not reveal significant changes. As shown by the XPS data (Table 2) [38], depending on the concentration of TiO_2_ supported on the zeolite, the obtained gold species were different. A higher relative concentration of Au metallic nanoparticles was evidenced for the YT5A sample with the smallest TiO_2_ crystallite size (Table 1) which most likely contributes to more intimate contact with immobilized gold. Thus, a strong interaction takes place which means an electron transfer between the support modified with TiO_2_ and the Au^3+^ species (used for impregnation), reducing them to metallic Au.

**Figure 8:**
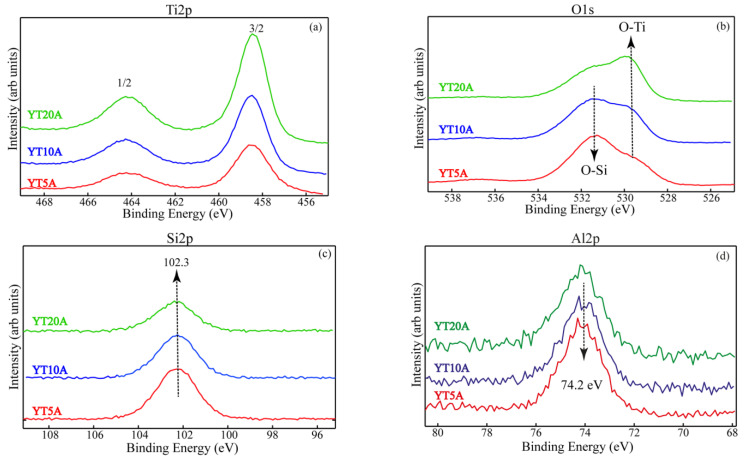
Ti2p (**a**), O1s (**b**), Si2p (**c**), and Al2p (**d**) superimposed high-resolution XPS spectra.

The surface chemistry of titanium, oxygen, silicon, and aluminum was evaluated for all the samples and the results are illustrated in Figure 8a–d. The two peaks recorded for Ti 2p (Figure 8a) are assigned to Ti^4+^ from Ti–O–Ti (lower binding energy) and Ti^4+^ from the isolated Ti–O–Si (higher binding energy) coordination [39]. The O1s high-resolution XPS spectra (Figure 8b) present two peaks: one is attributed to the O-Si band from zeolite support (~532.5 eV), and another one, with variable intensity from lower binding energy (~531 eV), indicates the presence of oxygen bonded to Ti in TiO_2_ species with octahedral coordination. For Si2p XPS spectra (Figure 8c), the peak located at BEs (102.3 eV) was assigned to Si-O from zeolite Y supports. Figure 8d confirms the presence of aluminum on the surface of the obtained samples. 

#### 2.2.5. Raman Spectroscopy

**Figure 9:**
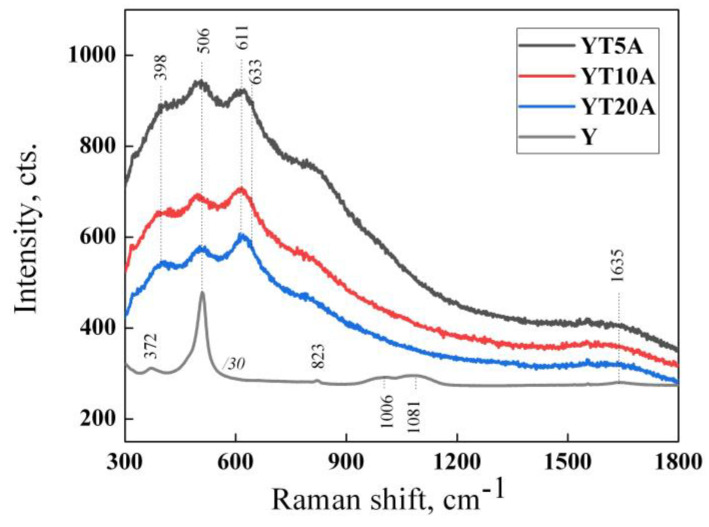
UV–Raman spectra of the YTxA samples (x = 5, 10, 20) and zeolite Y.

The UV–Raman spectral features of zeolite Y at 372, ~500, 1006, and 1081 cm^−1^ are masked by the stronger vibration modes of the framework titania [40] as rutile polymorph (611 and 826 cm^−1^ due to A_1g_ and B_2g_ modes [41]). The anatase phase is also depictable in Figure 9 by the weaker shoulders at 398 cm^−1^ (B_1g_) and 633 cm^−1^ (E_g_) [41]. 

Since the intensity of the band located at about 500 cm^−1^ decreases with TiO_2_ loading, it points out that the surface of the Y zeolite is better covered by TiO_2_.

#### 2.2.6. UV–Vis Absorbance Spectroscopy

**Figure 10:**
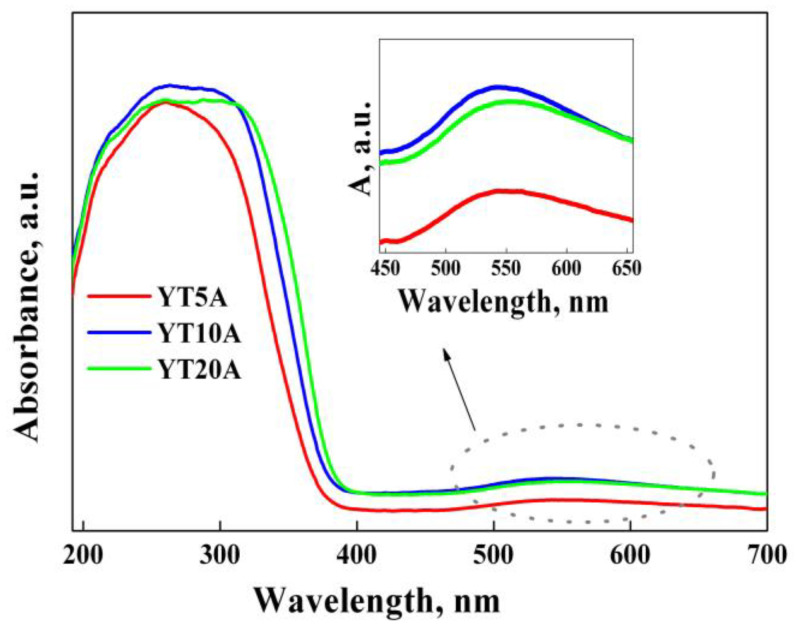
UV−Vis absorption spectra of Au–Ti photocatalysts.

The optical properties of the synthesized materials were investigated by UV–Vis absorption spectroscopy and the obtained spectra are shown in Figure 10. 

An intense absorption band in the UV range was obtained for all the photocatalysts, associated with tetrahedral Ti species (~220 nm), octahedral Ti-oxide species (~260 nm), and TiO_2_ as agglomerations on the zeolite surface (>300 nm) [42,43]. Furthermore, a red-shift of the absorption bands with titania amount was shown, corresponding to an increase in TiO_2_ particle size, supported by the values obtained using the Scherrer equation (Table 1). It can be explained by the quantum size effect that emerges for TiO_2_ species with particle size < 10 nm [44]. Another band absorption with a maximum of around 550 nm was observed in the visible domain, corresponding to the local surface plasmon resonance effect of gold nanoparticles [45]. The highest intensity of this absorption band was obtained for the photocatalyst with a TiO_2_ content of 10%. So, this concentration can be considered as optimal for ensuring the interaction between titania with gold species. Consequently, the intensity of the Au plasmonic effect increased as follows: YT5A < YT10A < YT20A. 

The indirect band gap energies were calculated using the Kubelka–Munk function by plotting [F(R)·hν]^1/2^ versus photon energy (eV). The obtained values (Table 1) suggest a decrease in the band gap energy after titanium immobilization compared to bulk TiO_2_ (3.2 eV) due to the high dispersion of titania [46,47]. A slight increase in the band gap was obtained by gold immobilization, as a result of plasmonic properties of gold nanoparticles that inject electrons into the conduction band of TiO_2_ (Burstein–Moss (BM) effect [48]). Therefore, sufficiently high energy is needed to allow the transfer of electrons to a higher free energy level. However, the band gap values calculated for the studied samples (Table 1) were considerably lower than those reported in the literature (3.54, 3.44 eV [35]).

#### 2.2.7. Photoluminescence Spectroscopy

**Figure 11:**
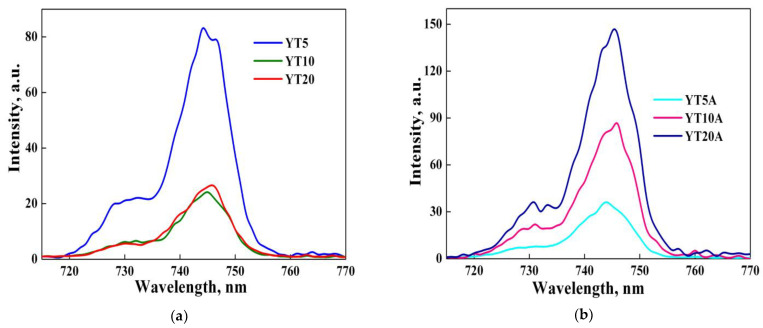
PL spectra of the YTx materials (**a**) and YTxA samples (**b**).

The photoluminescence spectra recorded for Ti-modified materials before and after gold immobilization are illustrated in Figure 11a,b.

It was noticed a decrease in the PL emission with TiO_2_ loading (Figure 11a), suggesting the enhancement of materials’ capacity to capture photogenerated electrons, improving e^−^/h^+^ separation. A better separation of electron–hole pairs in the case of higher TiO_2_ content, ultimately means better photocatalytic properties. After gold immobilization, different behaviors were noticed (Figure 11b) depending on the type of gold species that resulted after interaction with TiO_2_ added in various concentrations. Thus, the lowest PL emission was obtained for the YT5A sample which has only metallic gold nanoparticles and Au^3+^ species on the surface, as XPS results showed (Table 2).

#### 2.2.8. H_2_-TPR Studies

**Figure 12:**
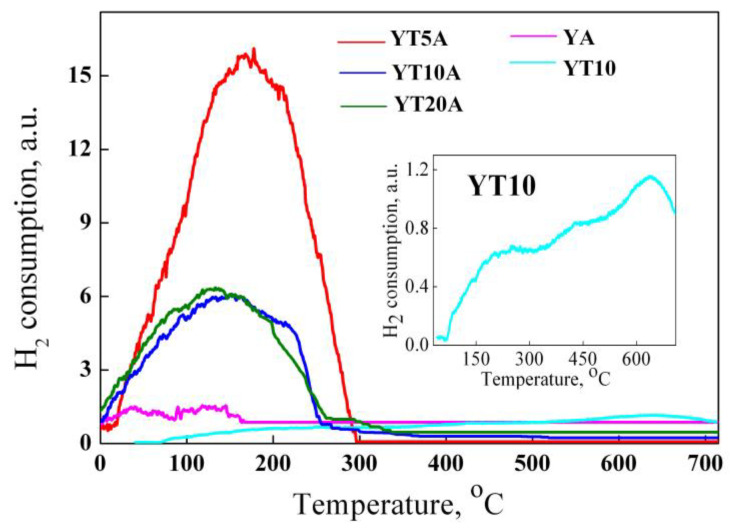
H_2_-TPR profiles of the obtained samples.

The influence of titania content on the materials’ reducibility was monitored by H_2_ temperature programmed reduction (H_2_-TPR). The TPR profiles show (Figure 12) broad reduction peaks. These were attributed to the reduction of gold cations (Au^+^ and Au^3+^), evidenced by XPS (Table 2), which interact with titanium species dispersed on supports. In the absence of Ti species, the reduction profile of the YA sample appeared as two peaks with a maximum of around 50 °C and 130 °C. The first has been assigned to the adsorption of hydrogen on zeolite Y [49] and the second to the reduction of Au^3+^ species to Au^0^ [50]. The 10% TiO_2_ loading on zeolite Y (sample YT10) can hardly react with H_2_. Two main very weak and broad signals at around 250 °C and 650 °C were evidenced by H_2_-TPR profiles (inset of Figure 12). The peak located at lower temperatures was attributed to the presence of subsurface oxygen, while the signal from higher temperatures was assigned to oxygen species that are strongly chemisorbed on the surface or to a possible reduction of Ti^4+^ to Ti^3+^ [51]. Therefore, Au–TiO_2_ interaction influenced the intensity and position of the reduction peaks. Two different profiles can be seen in Figure 12 for samples with different concentrations of titanium oxide. The highest peak with one maximum at 160 °C was obtained for the YT5A sample with the highest Au^3+^ species on the surface (Table 2). Similar TPR profiles were obtained for YT10A and YT20A samples except for one shoulder at around 100 °C (of the first) and another at 240 °C observed for the last one. The difference between these samples is the loading and nature of titanium species. Generally, the weak interaction (Au cations with TiO_2_ species) leads to reduction peaks at low temperatures, while a stronger interaction (Au with Ti–O–Si species) leads to reduction at higher temperatures [52]. 

The shift to lower temperature can be seen for both the YT20A sample (Figure 12). More significant shoulders of YT20A and YT10A reduction profiles can be assigned to the adsorption of hydrogen (the first) and hydrogen spillover (the second). In fact, metallic gold plays an important role in hydrogen spillover and thus reactive atomic hydrogen can directly diffuse over the solid surface and react with titanium oxide [53]. Furthermore, the hydrogen migration over non-reducible supports such as zeolites [54] is mediated by H· species which interact in particular with defect sites of this [55]. So, the defects’ abundance of support enhances the hydrogen diffusion and spillover phenomenon depending on their availability. The surface vacancies are the effect of titania loading, dispersion, and interaction with the surface of zeolite support. Regarding titanium species reduction, no characteristic peak was evidenced for all the samples, probably due to the high dispersion of these species, their high interaction with porous support, and the protective effect of gold nanoparticles, as it was suggested in a previous study [56].

#### 2.2.9. CO_2_-TPD Results

**Figure 13:**
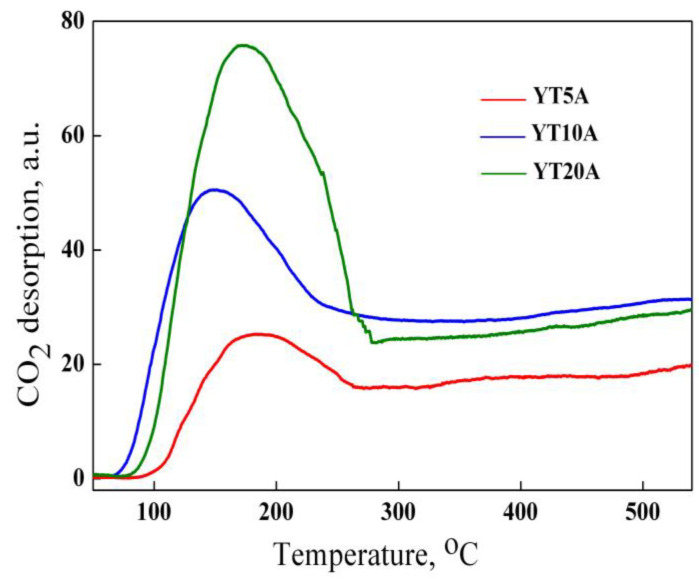
CO_2_-TPD profiles of the studied materials.

The CO_2_-temperature programmed desorption (TPD) profiles recorded for the samples showed a similar temperature (around 180 °C) of CO_2_ desorption peak for YT20A and YT5A samples (Figure 13) and a shift to a slightly lower temperature (150 °C) for YT10A sample. The peaks recorded in the thermal desorption spectrum are attributed to different bonds of CO_2_ to the regular sites on the surface. Desorption profiles show one peak for all the samples based on zeolite Y, indicating one kind of CO_2_ interaction (weak for YT10A, weak-moderate for YT5A and YT20A samples).

CO_2_-TPD results reflect also the increase in CO_2_ adsorption with titania loading. Therefore, the amount of TiO_2_ influences the number of sites, but also their strength. A different number of oxygen vacancies could be created by the immobilization of TiO_2_ on zeolite Y, as a result of the interaction between TiO_2_ and support. The electron pair remained in the vacancy after oxygen defect formation at the interface of TiO_2_ and SiO_2_ moved toward the neighboring Ti atoms [57].

#### 2.2.10. Adsorption Studies

It is well known that adsorption is an effective method for removing several pollutants from wastewater [58]. On the other hand, in photocatalytic degradation of organic molecules from wastewater, adsorption, and photocatalytic reactions occur simultaneously and have a synergistic effect on efficiency [59]. It was previously reported [20] for the case of the YT10A sample that in the first 3 h, the variation of AMX concentration in solution is strongly influenced by adsorption. After this period of time, the significant decrease in AMX concentration can be attributed to the photocatalytic process. In the photocatalytic degradation of AMX, adsorption can be considered a rate-determining step [47].

**Figure 14:**
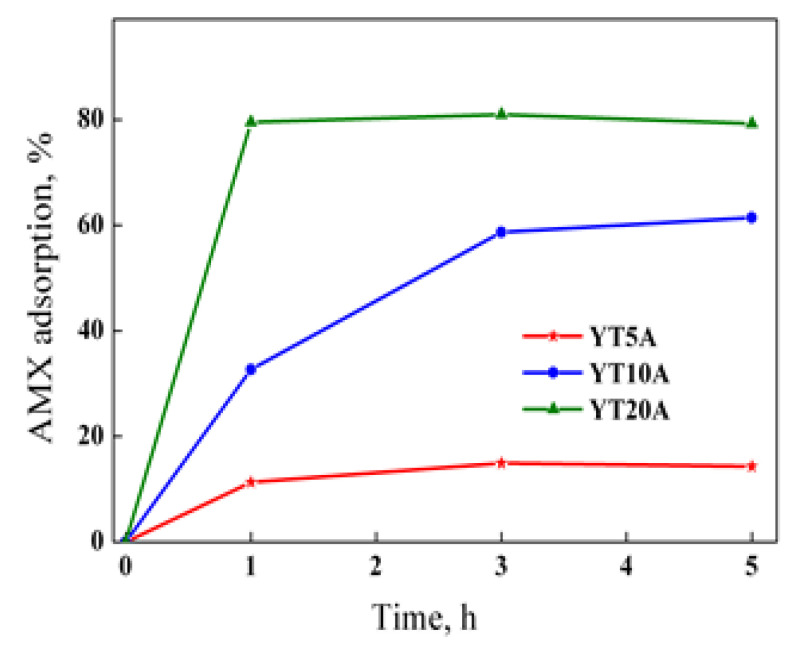
AMX adsorption capacity of the prepared materials.

The adsorption studies of AMX on the photocatalysts was evaluated in dark conditions. The obtained results are presented in Figure 14.

Figure 14 revealed a significant increase in AMX adsorption in the first hour. After that, the variation is insignificant, tending to a level reached after three hours for all samples. The adsorption capacity increased with titania content, such as the best results were obtained for the YT20A sample. It is observed that the maximum and minimum adsorption capacities were obtained for samples with the highest and lowest TiO_2_ loading. The increase in adsorption with TiO_2_ loading was attributed to the created defect sites that enhanced the surface basicity, as was demonstrated by CO_2_-TPD results (Figure 13). Furthermore, it seems that the weak-moderate character of the basic sites in the case of samples YT10A and YT20A favors the adsorption capacity of weakly acidic amoxicillin molecules.

**Figure 15:**
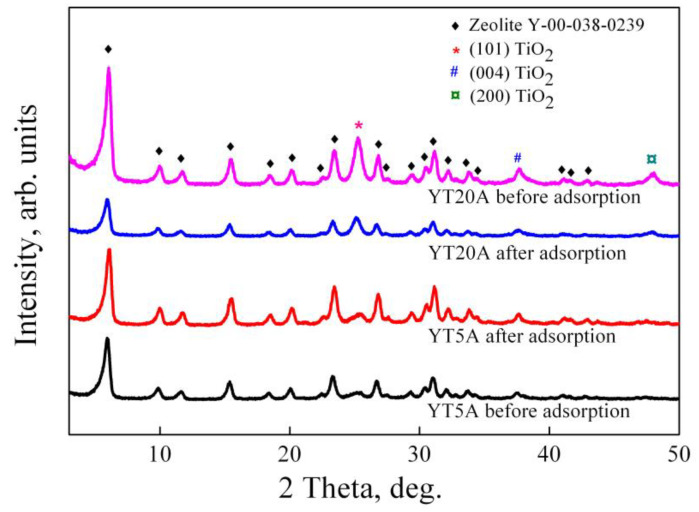
Wide-angle X-ray diffraction patterns of the samples before and after adsorption of amoxicillin.

The synthesized samples show a high stability, as was suggested by XRD measurements (Figure 15) which indicated the same pattern of materials before and after adsorption process. However, in the case of YT20A sample, a considerable decrease in the intensity of diffraction peaks was found. This result may be associated with the decrease in crystallinity and may be due to the presence of amoxicillin molecules in a larger amount on the zeolitic structure of the photocatalyst [60]. The results presented in Figure 14 indicate the highest adsorption capacity for this sample. 

#### 2.2.11. Photocatalytic Activity

**Figure 16:**
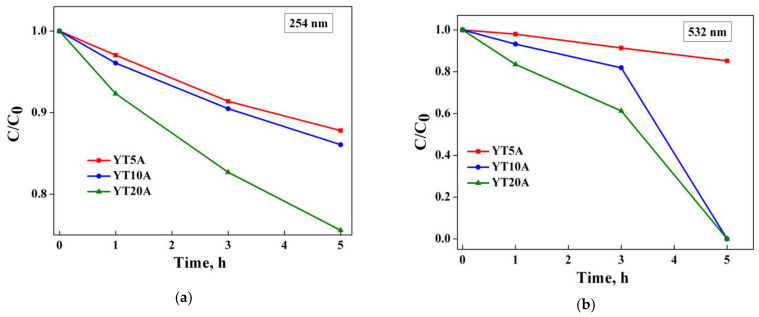
Photocatalytic degradation of amoxicillin under UV (**a**) and visible light irradiation (**b**) using the Au–Ti materials.

The degradation of AMX under UV and visible light irradiation (λ = 254 nm and λ = 532 nm) using the obtained photocatalysts showed the effect of TiO_2_ loading on the process efficiency. The obtained results are shown in Figure 16a,b.

It has shown a direct dependence between the photocatalytic process and adsorption capacity (Figure 14), highlighting the importance of providing pollutant molecules on the surface of the photocatalytic material, close to the activated sites under irradiation [47]. Thus, the photocatalytic properties of the synthesized materials increased with the TiO_2_ loading, both under UV and visible light irradiation.

It is worth discussing the exceptional results obtained in the case of samples with 10 and 20% TiO_2_ under visible light irradiation. As can be seen from Figure 16b, the total degradation of amoxicillin was achieved after 5 h of irradiation. This behavior can be explained by the presence of metallic gold nanoparticles Au NPs and additionally, gold nanoclusters Au NCs (see Table 2) which act as e^-^ donors under visible light irradiation. 

Both Au NPs and Au NCs have a key role in the photocatalytic performance of the synthesized materials, but each act differently. Thus, under the effect of visible irradiation, Au NPs exhibit surface plasmonic effect leading to the generation of hot electrons that are injected directly into the conduction band of TiO_2_. In addition to this aspect, the Schottky barrier that forms at the interface between the metallic gold nanoparticles and TiO_2_ prevents the return of the injected electrons, leading to a better separation of the e^−^/h^+^ species, which is desirable in photocatalysis [61]. In the case of photoactivated Au NCs, known as having abundant unsaturated active sites [62], there is a discrete e^-^ transition from the LUMO to HOMO energy levels and further, to the conduction band of TiO_2_ [61].

In the case of UV light irradiation, when only TiO_2_ is activated, the metallic gold species act as e^-^ trappers which would imply an improvement of the photocatalytic activity due to the separation of charge carriers [20,61]. However, the photocatalytic performances obtained are lower than in the case of visible irradiation (Figure 16). The same behavior is also discussed in other studies reported in the literature [63] and is attributed to the low capacity of Au NCs to accept electrons photogenerated by TiO_2_ [64] and to their low bandgap (1.3–1.4 eV) that allows activation only under visible irradiation [65,66].

#### 2.2.12. Kinetic Studies

**Figure 17:**
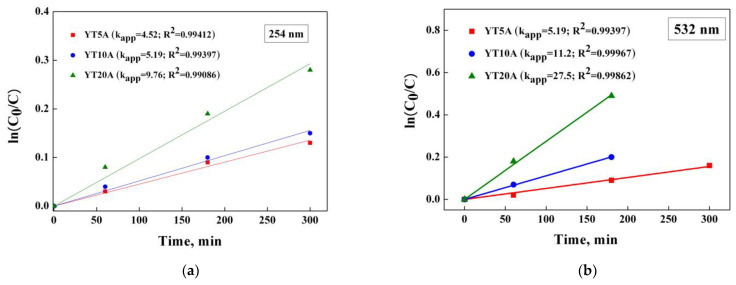
Kinetic and apparent rate constant k_app_ values (×10^−4^ min^−1^) of AMX photocatalytic degradation under UV (**a**) and visible light irradiation (**b**).

The photocatalytic degradation of amoxicillin by Ti–Au synthesized materials was studied using a pseudo-first-order kinetic model, expressed as ln (C_0_/C) = k_app_t. The kinetics results obtained under UV and visible light irradiation are presented in Figure 17a,b.

The high values of linear regression coefficients (R^2^) obtained for the kinetic plots for all the synthesized materials confirm the pseudo-first-order type of photocatalytic reactions, as was previously reported [67]. The calculated values of the apparent rate constant (k_app_) are exposed in Figure 17. It can be seen the increasing of k_app_ values with titania content. The effect is more significant under visible light due to the surface plasmon resonance (SPR) effect of gold in interaction with supported titania [68].

#### 2.2.13. Toxicity Assessment

**Figure 18:**
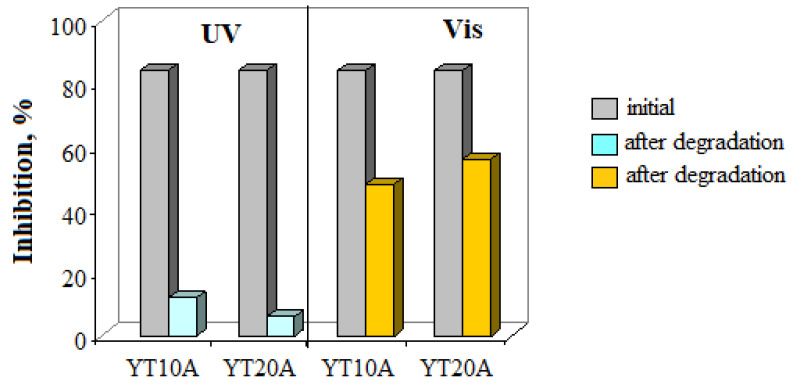
Changes in bacterial (*S. aureus)* inhibition of AMX solution after photocatalytic reactions under UV and visible light irradiation.

Toxicity assessment, before and after photocatalytic reactions, was conducted to evaluate the amoxicillin degradation and formation of non-toxic intermediates in terms of antimicrobial effect. The results are exposed in Figure 18 and show a decrease in inhibition percentage compared to the initial amoxicillin solution. Furthermore, it can be observed that irradiation conditions influence the nature of AMX degradation products and consequently, their toxicity. The toxicity results highlight the lowest residual toxicity under UV irradiation and a higher one in the case of visible light conditions. These results may be the effect of different reaction mechanisms for these photocatalysts under UV or visible light.

## 3. Conclusions

High-performance photocatalysts were synthesized by the processing of aluminosilicate gel in order to obtain zeolite Y with a very well-organized structure and further, modified with TiO_2_ and gold species by impregnation. Aging of aluminosilicate gel in static conditions and mixing the precursors required for the synthesis gel under magnetic stirring leads to the obtaining of highly crystallized zeolite Y with homogeneous morphology, desirable properties in tailoring high-performance photocatalysts. Different TiO_2_ loadings were used (5, 10, 20%), and was studied their interaction with the subsequently immobilized gold species. The main important changes that appeared in the structural, textural, morphological, and optical properties of materials were discussed. An increase in anatase crystallite size was noticed with TiO_2_ loading and a different reducing capacity of Au^3+^ species. XPS results showed the production of only metallic gold nanoparticles for the YT5A sample, while for the YT10A and YT20A samples, intermediate gold states of the Au^+1^ type, as well as clusters, were also obtained. PL spectroscopy showed that a larger amount of TiO_2_ led to a delay in the recombination capacity of the photogenerated electron–hole pairs, which is desirable in photocatalysis. Additionally, an improvement in the adsorption capacity of the organic pollutant (amoxicillin) was obtained by increasing the amount of TiO_2_, due to the high number of basic sites on the surface of the material. All these properties are responsible for the photocatalytic results obtained in the degradation of amoxicillin. Thus, the best performances were obtained for the YT20A sample with 20% TiO_2_, achieving a total degradation of amoxicillin under visible irradiation after 5 h. The plasmonic effect of gold nanoparticles also has a contributing role in the case of visible light irradiation. It was demonstrated that the materials synthesized in the present work have the ability to turn amoxicillin into products with much lower toxicity, both in UV and visible light.

## 4. Materials and Methods

### 4.1. Materials

#### 4.1.1. Chemicals

To obtain the aluminosilicate gel, we used sodium silicate solution (26.5 wt.% SiO_2_, 10.6 wt. % Na_2_O, Sigma Aldrich, Darmstadt, Germany) and sodium aluminate (NaAlO_2_, Sigma Aldrich) as silica and alumina sources, respectively. To provide the strong basic medium necessary for assembling structural units of zeolite Y, NaOH (98 wt.%, Lach-Ne) was chosen. Titanium (IV) n-butoxide (99%, Acros Organics, Geel, Belgium) and gold chloride (HAuCl_4_aq, purum 51.5% Au brown, Fluka, Buchs, Switzerland) were used as precursors for the modification of zeolite Y with TiO_2_ and Au species, respectively.

#### 4.1.2. Sample Preparation

**Figure 19:**
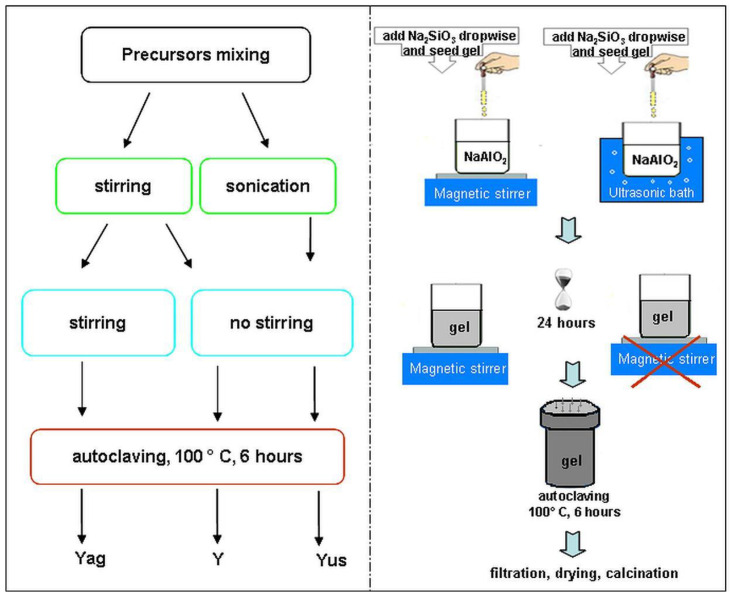
Schematic representation of the various treatments of aluminosilicate gel during the synthesis of faujasite Y.

The synthesis of zeolite Y was used as support for obtaining the photocatalytic materials and was carried out by a hydrothermal method in two steps that involves the use of seed gel obtained first [34]. In the second step, aluminosilicate gel with the final molar ratio 0.66Na_2_O:0.21Al_2_O_3_:SiO_2_:19.1H_2_O, was prepared and the resulting seed gel was added. This mixture was aged for 24 h at room temperature and further, 6 h at 100 °C in a Teflon-lined autoclave. The formation of the aluminosilicate gel and its evolution during the zeolitization process was described in detail in a study recently reported by our group [30]. In order to obtain the aluminosilicate support with suitable properties for photocatalysis, the treatment of the corresponding gel resulting from mixing sodium silicate with sodium aluminate was completed differently. As suggested in Figure 19, the method of mixing the precursors (stirring vs sonication), and the presence or absence of agitation during the aging of aluminosilicate hydrogel were varied. The zeolite powders thus obtained were denoted Yag, Y, and Yus.

Further, TiO_2_ and Au were immobilized on zeolite Y powder obtained by the second route (according to Figure 19) by impregnation method using the corresponding precursor. Firstly, TiO_2_ was supported on zeolite in different concentrations (5 wt.%, 10 wt.%, 20 wt.%) from an alcoholic solution of titanium (IV) n-butoxide. To eliminate traces of organics from the precursor, the samples were calcined in air at 600 °C for 8 h. Secondly, 1 wt.% gold was added on Ti-modified zeolite Y from an aqueous solution of HAuCl_4_. Chlorine was removed by washing with Na_2_CO_3_. Finally, the samples were washed with deionized water, dried at room temperature until the next day, and further at 120 °C for 6 h. The materials thus synthesized were labeled YT5A, YT10A, and YT20A.

### 4.2. Methods of Characterization

Wide-angle X-ray diffraction (XRD) patterns of the samples were recorded using a Rigaku Ultima IV diffractometer (Rigaku Corp., Tokyo, Japan) with Cu Kα (λ = 0.15406 nm). TiO_2_ crystallite size was calculated according to Scherrer’s formula: D = k·λ/ (FWHM)·cos(θ) along the (101) direction, where λ = wavelength of the Cu Kα radiation (1.54056 Å), FWHM = full width at half maximum of the intensity vs. 2θ profile, θ = Bragg’s diffraction angle and k = 0.9 (shape factor).

N_2_ physisorption analysis was performed for textural characterization of the samples using a Micromeritics ASAP 2020 analyzer (Norcross, GA, USA).

Morphological investigations of zeolite Y before and after modification with Ti and Au active species were performed by means of a scanning electron microscope (ZEISS EVO LS10 SEM, Oberkochen, Germany).

X-ray photoelectron spectroscopy was used to analyze the main species on the surface of the materials using an XPS—PHI Quantera equipment (Ontario, ON, Canada) with a monochromatized Al Kα radiation (1486.6 eV). The overall energy resolution was estimated at 0.6 eV by the full width at half-maximum (FWHM) of the Au4f7/2 photoelectron line (84 eV).

UV–Raman spectra of the samples were recorded by means of a LabRam HR800 spectrometer (Horiba France SAS, Palaiseau, France). Catalysts were excited with a 325 nm line through an x40/0.47NUV objective from Olympus Corporation, Tokyo, Japan. The experimental setup (UV laser and rejection filter) prevented recording Raman spectra at lower wavenumbers.

The UV–Vis diffuse reflectance spectra of the photocatalysts were recorded in the range of 200–700 nm using a JASCO V570 spectrophotometer (Tokyo, Japan).

An FLSP 920 spectrofluorimeter (Edinburgh Instruments, Livingston, UK) with a Xe lamp as an excitation source (λ_exc_ = 550 nm) was used to record the photoluminescence spectra of the samples between 570 and 800 nm. The excitation and emission slits were 10 nm for all measurements.

For the temperature-programmed reduction of the samples by hydrogen (H_2_-TPR), a ChemBET 3000-Quantachrome (Boynton Beach, FL, USA) coupled with a thermal conductivity detector (TCD) was used. The experiments were carried out in a flow system using Ar (70 mL/min) and 5% volume H_2_ over 50 mg photocatalyst. H_2_-TPR profiles were recorded up to 850 °C, with a heating rate of 10 °C/min. A silica gel column was used for the optimal operation of the thermal conductivity detector.

The adsorption properties for CO_2_ were measured by TPD of CO_2_ with a Quanthachrome ChemBET 3000 apparatus (USA) equipped with a thermal conductivity detector (TCD). Before adsorption, the samples (50 mg) were pretreated with a helium flux from room temperature up to 300 °C and kept for 1 h (heating rate of 10 °C/min). Then, the samples were cooled at 100 °C and saturated with a CO_2_ stream (30 mL/min). After baseline stabilization, the temperature of the reactor was increased by 10 °C/min until all the CO_2_ was desorbed. Desorbed CO_2_ amounts were calculated from the area of the obtained peaks. 

The adsorption and photocatalytic experiments were carried out under stirring in a dark room at a constant temperature of 30 °C by adding 20 mg of the photocatalyst 10 mL aqueous solution of amoxicillin, AMX (30 mg/L). For the photocatalytic tests, a halogen lamp (2 × 60 W) was used with a filter for λ = 254 nm and a DPSS-532-100 laser (Apel Laser, Bucharest, Romania) for visible light (λ = 532 nm). The UV or visible light irradiation was started after stirring the reaction mixture for 30 min in the dark in order to allow the AMX adsorption on the photocatalysts’ surface. At given intervals of time (1, 3, and 5 h of irradiation), 3 mL of suspension was taken out and filtered using a Millipore syringe filter of 0.45 μm in order to remove the photocatalyst from the suspension. Further, the filtered solution was spectrophotometrically analyzed by means of the same JASCO V570 UV–Vis spectrophotometer, reading the maximum absorbance of AMX molecules (λ = 230 nm). Finally, the evaluation of AMX degradation was expressed as C/C_0_, where C is the concentration of the solution taken out at time t (1, 3, or 5 h) and C_0_ is the initial concentration, at t = 0. The relation ln(C/C_0_) = k_app_t was used to obtain the apparent rate constant (k_app_), considering that the AMX degradation process is a first-order reaction. 

Toxicity assessment, before and after photocatalytic reactions, was conducted to evaluate the amoxicillin degradation and formation of non-toxic intermediates in terms of antimicrobial effect. For this purpose, the antibacterial activity was performed by diffusimetric method using disk and spot inoculation on agar Mueller Hinton medium (Scharlau, Spain) with the composition (g/L: 17.5, peptone; 1.5, starch; 2.0, meat infusion solids). Bacterial suspension of *Staphylococcus aureus* (with concentration of 1−3 × 10^8^ CFU/mL) was used to inoculate the agar media, followed by the application of a volume of 10 µL of the tested solution. Subsequently, plates were incubated for 24 h at 35 °C. The antibacterial effect was evaluated by observing the presence and measuring the clear halo developed around the inoculation area.

## Figures and Tables

**Table 1:** Textural and optical properties of the samples.

Sample	BET Surface Area (m^2^/g)	Vpore (cm^3^/g)	Pore Size (nm)	TiO_2_ Crystallite Size (nm)	Band Gap Energy (eV)
YT5	850	0.102	1.6	9.9	3.09
YT5A	856	0.104	1.6	10.0	3.22
YT10	678	0.107	1.8	10.2	3.17
YT10A	699	0.109	1.8	10.3	3.20
YT20	507	0.114	2.0	12.1	3.14
YT20A	572	0.154	2.0	12.3	3.15

**Table 2:** XPS data: binding energies (BEs) and the quantitative assessment.

Sample	Binding Energy (eV)				Au Chemical Species Rel. Conc.			
	Au4f/2 Metallic nps	Au4f/2 Clusters	Au4f/2 Au+	Au4f/2 Au3+	Au Metallic nps	Clusters	Au^1+^	Au^3+^
YT5A	83.3	84.3	85.3	86.7	56.1	-	-	43.9
YT10A	83.3	84.3	85.3	86.7	44.7	18.8	18	18.6
YT20A	83.3	84.3	85.3	86.7	45.7	19.2	16	19

## Data Availability

The data presented in this study are available on request from the corresponding author.
